# Priorities to Support Care Partners of People Living with Dementia: Results of a Modified Delphi Process

**DOI:** 10.1002/alz.087792

**Published:** 2025-01-09

**Authors:** Walter D Dawson, Sherril B Gelmon, Jenn Hollandsworth Reed

**Affiliations:** ^1^ Global Brain Health Institute, San Francisco, CA USA; ^2^ OHSU‐PSU School of Public Health, Portland, OR USA; ^3^ Portland State University, Portland, OR USA; ^4^ NIA‐Layton Aging & Alzheimer’s Disease Center, Portland, OR USA

## Abstract

**Background:**

Alzheimer’s disease and related dementias (ADRD) place substantial burdens on care partners. The need to better support ADRD care partners through policy actions is high, specifically to enhance existing services and reduce inequities experienced by historically underserved communities.

**Method:**

Between July and September 2023, a modified Delphi process was employed to determine policy and program priorities for supporting Oregon‐based care partners. Representatives of organizations serving people living with ADRD and their care partners, as well as organizations serving selected historically and currently underrepresented communities, were invited to participate (N = 40). Prospective participants created and categorized a preliminary list of ideas during an initial meeting. The eight thematic categories (Care Partner Supports, Programs/Services, Information Resources, Care Coordination, Data Systems/Technology, Policy, Funding/Financing, and Professional Education/Workforce Development) framed the three surveys for the Delphi process. In survey 1, respondents received the list of ideas and were invited to generate up to five additional priority ideas per category. In survey 2, participants reviewed the revised list and rated each idea on a five‐point scale of importance. In survey 3, participants were presented with the mean importance score for each idea and then indicated five top priorities for each thematic category.

**Result:**

The 15 ideas with the highest weighted scores were each endorsed by at least 60% of survey 3 respondents, and were arrayed across six of the thematic areas: Care Partner Supports = 4, Programs/Services = 3, Funding/Financing = 3, Information Resources = 2, Care Coordination = 2, and Professional Education/Workforce Development = 1.

**Conclusion:**

The use of the Delphi process facilitated input from individuals with different perspectives, and gave all participants’ contributions equal weight, achieving a balance of power and minimization of bias that might not have occurred through methods such as in‐person discussion and debate. This process identified specific and actionable priorities to better support Oregon care partners through program development and policy action, and may have applicability elsewhere.

